# Sellers of unpasteurized animal milk in unregulated markets: a key factor for human brucellosis control in El-Oued province, Algeria

**DOI:** 10.11604/pamj.2025.51.97.48308

**Published:** 2025-08-19

**Authors:** Bachir Khezzani, Mile Bosilkovski, Marija Dimzova, Jadranka Nikolić, Borislava Chakarova, Ilia Tsachev, Magdalena Petrova Baymakova

**Affiliations:** 1 Department of Biology, Faculty of Natural Sciences and Life, El Oued University, El Oued, Algeria,; 2 Laboratory of Biology, Environment and Health, Faculty of Natural Sciences and Life, El Oued University, El Oued, Algeria,; 3 University Hospital for Infectious Diseases and Febrile Conditions, Medical Faculty, Ss Cyril and Methodius University, Skopje, Republic of North Macedonia,; 4 University Clinical Hospital Mostar, School of Medicine, University of Mostar, Mostar, Bosnia and Herzegovina,; 5 Department of Hygiene, Epidemiology, Microbiology, Parasitology and Infectious Diseases, Faculty of Medicine, Trakia University, Stara Zagora, Bulgaria,; 6 Department of Microbiology, Infectious and Parasitic Diseases, Faculty of Veterinary Medicine, Trakia University, Stara Zagora, Bulgaria,; 7 Department of Infectious Diseases, Military Medical Academy, Sofia, Bulgaria

**Keywords:** Animals, brucellosis, humans, public health, unpasteurized milk, zoonoses

## To the editors of the Pan African Medical Journal

Brucellosis is a highly contagious zoonotic infection affecting livestock and human beings [1]. The pathogen is a group of bacteria belonging to the genus *Brucella* [2]. Out of the identified species *(Brucella abortus, Brucella canis, Brucella melitensis, Brucella suis)* are responsible for human brucellosis outbreaks [3]. It is mainly acquired through the consumption of undercooked animal products, inhalation of contaminated aerosols, or direct contact with infected animals [4]. Owing to its danger to public health and countries' economies, *Brucella* is classified by the Centers for Disease Control and Prevention (CDC) as a category B biological warfare pathogen [5].

In humans, brucellosis is a treatable disease with many challenges [6]. Although governments have invested considerable financial and human resources in research on developing treatments for brucellosis, deployed efforts have yielded few results [6]. However, no effective vaccine has yet been licensed for human brucellosis [2].

**Figure 1:**
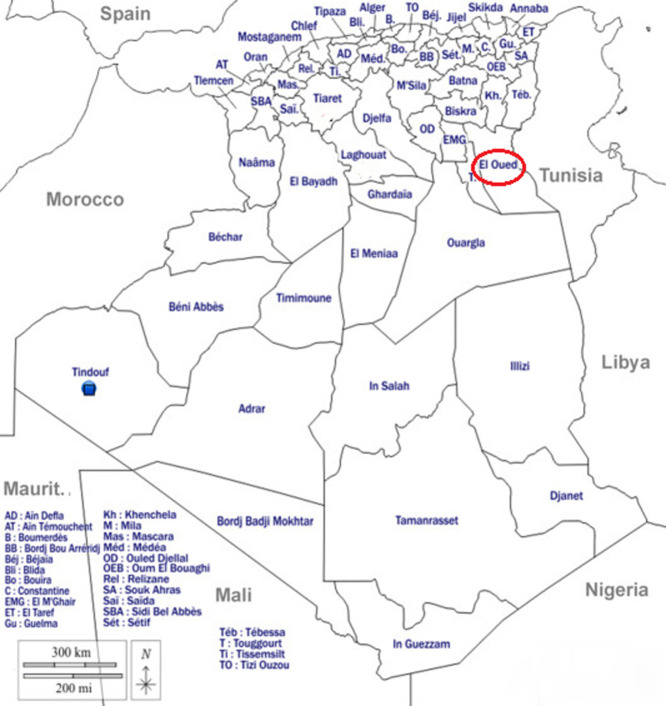
geographic location of El-Oued province, Algeria (in red)

El-Oued is a province located in Southeastern Algeria (Figure 1). According to Khezzani *et al*. [7], animal brucellosis has caused a wide outbreak in this province, especially in goat herds. In addition, the status of human brucellosis is the same- Khezzani *et al*. [8] reported 1.832 confirmed cases from 1998 to 2018. Despite the efforts of the local health authorities, the disease is still widespread in this province, where the latest statistics showed that from 2019 to 2023, more than 536 additional cases of animal brucellosis were reported, and 882 new cases of human brucellosis (2019-2024).

For this reason, ethical considerations reported by Khezzani *et al*. [2] effectively contributed to this situation; however, local investigations carried out by health authorities confirmed that unpasteurized milk consumption is the leading cause of the brucellosis outbreak in this province. This point of view is supported by many reports and investigations that concluded that consuming raw milk or its derived products was the principal cause of brucellosis infection, especially in rural areas [4]. In Algeria, a review conducted by Moustafa Kardjadj [5] reported that consuming raw milk and its derivatives has been implicated in 85% of human infections. Raw milk is widely consumed by the local population in this province. This behavior is driven by the prevailing belief that pasteurization causes milk to lose its natural taste and nutritional value. Furthermore, such behavior becomes more dangerous if this milk is used for infant nutrition.

In general, milk is obtained from popular markets. A group of shortcomings can be observed in the milk supply chain, which we summarize as follows: (i) raw milk is primarily produced from goats, because small farmers rarely have bovines; (ii) milk production is a secondary activity that complements agriculture and is the principal activity of most small farmers; (iii) these animals are likely to be infected with brucellosis because they are not generally subjected to veterinary health care. Furthermore, many studies reported that goats are more susceptible to brucellosis than sheep and bovines, which was confirmed by the study of Khezzani *et al*. [7] in the case of El Oued province; (iv) milk does not undergo pasteurization and is not subject to hygiene standards; (v) milk is packed in plastic bottles (soft drinks, mineral water, etc.) and often collected in public spaces; (vi) in popular markets, milk is exposed to light and high temperatures that may exceed 45.0°C.

Health authorities have become aware of the role of unpasteurized milk in transmitting brucellosis; therefore, the trade directorate (Algeria) issued a decision prohibiting the sale of unpasteurized milk, regardless of its source and preservation method.

Most studies in this field confirm that the consumption of raw milk is not only a risk factor for brucellosis transmission but also an important factor in transmitting a broad group of dangerous pathogens, such as *Campylobacter spp., Candida albicans, Candida krusei, Coxiella burnetii, Escherichia coli*, Hepatitis A Virus (HAV), Hepatitis E Virus (HEV), *Listeria monocytogenes, Mycobacterium bovis, Nocardia asteroides, Salmonella typhimurium, Staphylococcus aureus*, Tick-borne encephalitis virus (TBEV), and *Yersinia enterocolitica*, Norovirus [9]. Additionally, the behavior discussed in this paper is not only found in the aforementioned province but has also been observed in many other places, including the provinces of Tissemsilt [1] in the country, as well as internationally in Azerbaijan, Iran, Rwanda, and Saudi Arabia [4].

Such behavior undermines the state's efforts to combat brucellosis. Thus, both the seller and buyer bear part of the responsibility for the human brucellosis outbreak by selling and consuming unpasteurized milk. As most sellers and consumers are unaware of this matter, awareness campaigns may be a decisive factor in reducing the spread of the disease. Awareness campaigns should promote the understanding that the pasteurization process does not reduce the nutritional value of milk or change its taste. Therefore, some simple strategies, such as boiling milk at home before consumption, are the most cost-effective ways to protect consumers, as *Brucella* is sensitive to temperature and may be killed at 60.0°C for 10 minutes [10]. Housewives should be a part of awareness campaigns programs because they play a significant role in brucellosis control through their involvement in food preparation and their potential to influence family health practices.

Local authorities must enhance surveillance strategies by intensifying monitoring patrols in the area's popular markets. Simultaneously, the trade directorate must identify a mechanism that allows small farmers to market their dairy products without contributing to the spread of brucellosis.

## Conclusion

Milk is an important nutrient for all age groups and can transmit a wide range of pathogens, including *Brucella*. The fight against the sale of unpasteurized milk in popular markets, as well as its consumption, is a key factor in controlling brucellosis in El Oued province (Algeria) and in all communities and regions where this behavior is prevalent.
